# Childhood stunting and associated factors among irrigation and non-irrigation user northwest, Ethiopia: a comparative cross-sectional study

**DOI:** 10.1186/s13052-021-01048-x

**Published:** 2021-04-26

**Authors:** Balew Sema, Muluken Azage, Mulat Tirfie

**Affiliations:** 1 Bahir Dar University Student Clinic, Bahir Dar University, P.O.Box 79, Bahir Dar, Ethiopia; 2 Department of Environmental Health, School of Public Health, College of Medicine and Health Sciences, Bahir Dar University, P.O.Box 79, Bahir Dar, Ethiopia; 3 Department of Nutrition and Dietetics, School of Public Health, College of Medicine and Health Sciences, Bahir Dar University, P.O.Box 79, Bahir Dar, Ethiopia

**Keywords:** Childhood stunting, Irrigation and non-irrigation user, Mecha District, North west, Ethiopia

## Abstract

**Background:**

Stunting is a critical public health problem of developing countries like Ethiopia. Different interventions like irrigation activity have been carried out by the government of Ethiopia to improve the nutritional status of the community. However, there is scanty of data on childhood stunting and its associated factors between irrigation user and non-irrigation user.

**Objective:**

To assess the magnitude of childhood stunting and its associated factors between irrigation and non-irrigation user in North Mecha District, Northwest Ethiopia.

**Methods:**

A community-based comparative cross-sectional study design was employed from October to November 2019. A systematic sampling was used to draw mothers with children age 6–59 months (582 irrigated and 582 non- irrigated household users). Data were collected using questionnaire and anthropometric measurement tools. Multivariable logistic regression was used to identify the predictors of stunting. Adjusted odds ratios with 95% CI were used to determine the degree of association between independent and outcome variable. A-*p*-value < 0.05 was used as cutoff point to declare statistically significant variables with the outcome variable.

**Results:**

The prevalence of childhood stunting (6–59 months) among irrigation users [32.8% at 95%CI [29.1%-36.7%]] was slightly lower than non-users [40.2% at 95%CI [[36.3%-44.2%]]]. However, the difference did not show significant variation. The odds of childhood stunting were higher among a child from a mother had no antenatal visit, a child whose age was between 12 and 47 months, a child from a mother who did not use water and soap always for washing hands, and a child who had fever.

**Conclusion:**

The prevalence of childhood stunting was high and did not show significant variation between irrigation and non-irrigation users. A child from mother had no antenatal visit, whose age was between 12 and 47 months, a mother who did not use water and soap always for washing hands, and who had fever were factors associated with higher child stunting. Thus, the identified modifiable factors should be strengthened to reduce stunting.

**Supplementary Information:**

The online version contains supplementary material available at 10.1186/s13052-021-01048-x.

## Introduction

Globally, stunting remains one of the major public health problems among children under 5 years of age [1]. It is an indicator of malnutrition in early childhood and is strongly associated with numerous short-term and long-term conditions [2, 3]. It is the best of overall indicator of children’s well-being and an accurate reflection of social inequalities. Use of stunting as a direct indicator of nutritional status have shifted focus away from considering a broad set of environmental and social determinants of child growth to a disproportionate emphasis on dietary determinants of linear growth [4]. It is the result of the complex interaction between the food we eat, our overall state of health, and the environment in which we live in [5, 6].

The burden of stunting is multidimensional which are related with retarded growth, behavior development, poor school performance, lower working capacity, losses in cognitive function, losses in income, decreases productivity, and increased likelihood of chronic disease, if not corrected, it can slow down economic growth and increase poverty levels [7–9]. In the past 20 years, 55.5 billion Ethiopian birr (1.51 billion USD) are estimated with the costs associated with child under nutrition, equivalent to 16.5% of Gross Domestic Product (GDP) [10–12].

In 2019, the prevalence of stunting in Ethiopia was estimated to be 37% [13]. In the same year, the percentage of stunting is higher in Amhara region compared to national figure (41%), indicating inappropriate child feeding practices in the region [13]. Stunting usually increases between age 6 and 23 months, and peaks at age 24–35 months due to different reasons.

Different intervention with national nutrition strategies, nutrition specific and nutrition sensitive intervention activities with focus on improving Infant and Young Child Feeding have been implemented to reduce chronic malnutrition. Increasing agricultural production through irrigation system to meet food security at household level is also one of the strategies to address the basic needs of the community. Studies on prevalence of childhood stunting so far have been conducted in general community [7, 14, 15], which are either irrigated or non-irrigated users. To the best of our knowledge, there is not specific study of the prevalence of childhood stunting and associated factors among irrigation and non-irrigation users. This study aimed to answer the following research questions: (1) Is the magnitude of childhood stunting differ between irrigation and non-irrigation users? and (2) Which factors do determine the occurrence of childhood stunting in North Mecha District, Amhara Region, Northwest Ethiopia? The findings from this study will help to irrigations users in identify the gap and maximize the potential of irrigation for their health benefits.

## Methods

### Study area and period

The study area is Mecha district which is located at 530 kms in Northwest direction of Addis Ababa, the capital city of Ethiopia, and 35 kms in the Southwest direction of Bahir Dar, capital city of Amhrara National Region State. It is one of the thirteen districts found in West Gojjam Administrative Zone. The district comprises three climatic zone; high land “Dega”, mid-altitude “Wena Dega” and lowlands “Kola”. The mean annual rainfall ranges from 1000 mm to 2000 mm. The district has 156,027 ha area, of which 72,178 ha are used for cultivation and about 1386 ha covered by water bodies. The district has 40 Kebeles (Kebele is the lowest administrative unit in Ethiopia) (10 irrigation users and others are non-irrigated). Irrigation users are all households in a Kebele have access to the irrigation system from Koga dam [16]. Based on the 2007 national census, the estimated population of the district in 2019, is about 303,208, of which 150,088 are males and 153,120 are females. The district has 10 health center and 38 health posts and 1 government hospital during the time of the study [17]. The district is one of the food surplus areas and the commonest staple foods are maize, millet, teff and barley [16]. The study was conducted from October to December in 2019,

### Study design and population

A community-based comparative cross-sectional study design was employed to assess the prevalence and its associated factors of childhood stunting. All mothers/care-takers with children age 6–59 months in both irrigation users and non-irrigation were source of population in the district. All mothers/care takers with children age 6–59 months in the randomly selected Kebele were study populations.

### Sample size determination

The adequate sample size for this study was determined using two-population proportion formula [n (in each group) = f (α, β) (p1q1 + p2q2) / (p1 - p2)^2^] with the following assumptions: f (α, β) =7.84, when the power = 80%, the level of significance = 5% and the prevalence of stunting among irrigating (P1) was 10% and non-irrigated (P2) was17% from previous study in Kenya [18], design effect of 1.5 and 5% of non-responses rate. Based on the above assumptions, a sample size of 1164 HHs (582 irrigation users and 582 non-irrigation users) was calculated for the study.

### Sampling technique and sampling procedure

First, Kebeles in the district were stratified based on irrigation status (irrigation users and non-irrigation users). Six Kebeles from each stratum were selected randomly and proportion to size allocation was made to determine the required households from each randomly selected Kebeles. Households from randomly selected Kebeles were selected using systematic sampling technique. Sampling interval was determined by dividing the total number of households in each Kebele with the allocated sample size. The schematic sampling procedures was attached as supplementary file (Supplement file 1).

### Data collection tools

Socio-demographic and other data were collecting by using structured questionnaire adapted from different standard questionnaires and literatures. The anthropometric data was collected by using WHO Recommendations for data collection, analysis and reporting on anthropometric indicators in children under 5 years old [19]. A vertical or horizontal measuring board reading a maximum of 175 cm and capable of measuring to 0.1 cm was used to take the height of a child. The child stands on the measuring board barefoot; have hands hanging loosely with feet parallel to the body, and heels, buttocks, shoulders and back of the head touching the board. The head was held comfortably erect with the lower border of the orbit of the eye being in the same horizontal plane as the external canal of the ear. The headpiece of the measuring board is then pushed gently, crushing the hair and making contact with the top of the head. Height is then read to the nearest 0.1 cm. For those with written evidence, date of birth was obtained from clinic cards, child health cards and immunization status certificates of the children, unless by asking mother/care takers age was determined.

### Operational definitions

Irrigation users are all households in a Kebele have access to the irrigation system whereas non-irrigation users refer household which did not have irrigation system. Improved water includes piped water on premises such as piped household water connection located inside the user’s dwelling, plot or yard, public taps or standpipes, tube wells or boreholes, protected dug wells, protected springs and rainwater collection. Unimproved drinking water sources include unprotected dug wells, unprotected springs, carts with small tank/drum, tanker trucks, surface water (river, dam, lake, pond, stream, canal, irrigation channels) and bottled water. Improved sanitation facilities are used by only one family and can include toilets connected to sewers or septic systems, water-based toilets that flush into pits, simple pit latrines with slabs, and ventilated improved pit latrines. Unimproved sanitation facilities include those shared by more than one household, flush/pour flush to elsewhere in the environment without proper waste water treatment, the use of buckets, hanging latrines, or pit latrines without slab coverings and open defecation.

### Data quality assurance

Pretest of the questionnaire, training and calipering of the weigh scale were done to assure data quality. The questionnaire was developed from previous literature and field pre-tested on 5 % of sample size was done. Before the actual data collection, training was given for data collectors and supervisors. During the training, the trainers give instructions on the questions to be asked, their meaning, how to ask them, and how to record the answers. Data collectors and supervisors were also trained using role play practices. The equipment that was used to measure the anthropometric variables was calibrated between five respondents for actual data collection by using a known weight material. At the end of every data collection day, each questionnaire was examined for its completeness and consistency by the principal investigator and pertinent feedback was given to the data collectors and supervisors. All completed Epi (survey solution software) collected data entered questionnaire was conducted daily and Examined by principal investigator.

### Data processing and analysis

Data were collected using Epi collect survey solution software and directly downloaded to Excel, exported to SPSS version 23. Data was cleaned before data analysis. The anthropometric data was exported to WHO Anthro plus software to computed individual level of nutritional status. Frequencies, proportions and chi-square were computed for description of the study population in relation to socio-demographic and other variables. Bivariate and multivariable analysis logistic regressions were done to determine the association between stunting and explanatory variables. Those independent variables whose *p*-value less than 0.2 at the bivariable regression were included in a multivariable logistic regression model. Adjusted odds ratio with 95% CI was calculated to measure the degree of association between stunting and explanatory variables. A-*p*-value < 0.05 was used as cutoff point to declared statistically significant variables with the outcome variable.

### Ethical consideration

The study was approved by the Ethics Review Board of College of Medicine and Health Sciences Board (CMHS/IRB 03–008), Bahir Dar University. A support letter from the University was written to the Amhara Regional Health Bureau. Permission and a letter of support for the study were then obtained from the Amhara Region Health Bureau and the Regional Health Bureau letter (//03/455) was given to the district health office to commence the study. Information related ethical issue was given for mothers/caretakers (since most of them are farmers and could not able to read and write) by reading its content attached together in the first part of the questionnaire. Each study participant was briefed on the objective of the study, and informed about the right to refuse to participate in the study or to discontinue at any time. Participants were also informed that all data was confidential and personal identifiers were not registered. Interview was conducted after the parents and/or legal guardians of the child participants on their behalf provided verbal consent to participate in the study.

## Results

### Socio-demographic and economic status of participants

Of 1164 Households (HHs) randomly selected to participate in the study, 582 HHs from each irrigation and non-irrigation users were included in the analysis. Sixty eight percent of the respondent were found at greater and equal to 30 years, 71.0% from irrigated and 65% from non-irrigated users. Seventy-six from (76.3%) irrigation users and 72.2% from non-irrigation users had greater than four family members. Above 80 % of mothers from irrigation users (83.0%) and non-irrigation (80.9%) were unable to read and write (Table 1).

**Table 1:** Socio-demographic and economic status of respondents in irrigation and non-irrigation users in Mecha district, Northwest Ethiopia, 2019

Characteristics	Irrigated		Non-irrigated		Total	Chi-square (p-value)
	Frequency	percent	Frequency	percent		
**Mother’s age**						
< 20 years	16	2.7	17	2.9	33 (2.8)	4.70 (0.095)
20–29 years	153	26.3	186	32.0	339 (29.1)	
≥ 30 years	413	71.0	379	65.1	792 (68.0)	
**Family size**						
≤ 4 members	138	23.7	186	32.0	324 (27.8)	9.85 (0.002)
> 4 members	444	76.3	396	68.0	840 (72.2)	
**Mother education**						
Unable to read and write	483	83.0	471	80.9	954 (82.0)	0.84 (0.36)
Able to read and write	99	17.0	111	19.1	210 (18.0)	
**Antenatal care visit**						
No visit	6	1.0	40	6.9	46 (4.0)	27.26(< 0.001)
1–3 visits	333	57.2	330	56.7	663 (57.0)	
> 3 visits	243	41.8	212	36.4	455 (39.1)	
**Frequency of PNC**						
No visit	484	83.2	512	88.0	996 (85.6)	13.5 (0.001)
1–2 visit	83	14.3	46	7.9	129 (11.1)	
> 3 visit	15	2.6	24	4.1	39 (3.4)	
**Time taken to reach the health facility**						
< 1 h	442	75.9	207	35.6	649 (55.8)	192.32(< 0.001)
≥ 1 h	140	24.1	375	64.4	515 (44.2)	
**Drinking water source**						
Improved source	438	75.3	430	73.9	868 (74.6)	0.29 (0.590)
Unimproved source	144	24.7	152	26.1	296 (25.4)	
**Latrine facility**						
Improved latrine	394	67.7	222	38.1	616 (52.9)	102.01 (< 0.001)
Unimproved latrine	188	32.3	360	61.9	548 (47.1)	
**Ways of Hand washing habits**						
Only with water	26	4.5	208	35.7	234 (20.1)	184.34(< 0.001)
Sometime with soap and water	479	82.3	344	59.1	823 (70.7)	
Always with soap and water	77	13.2	30	5.2	107 (9.2)	

Forty-one percent of mothers from irrigation users and 36.4% of mothers from non-irrigation users had greater than three antenatal care (ANC) visit during their pregnancy. Eighty-three percent of mothers from irrigation users and 88.0% of mothers from non-irrigation users had no postnatal care (PNC) visit after birth. Regarding health institution location, 24.4% of mothers from irrigation users and 64.4% of mothers from non-irrigation users took one hour and above (Table 1).

Two-third of HHs (75.3%) from irrigation users and 73.9% of HHs from non-irrigation users had improved water source. Sixty-eight of HHs (67.7%) from irrigation users and 38.1% of HHs from non-irrigation users had improved latrine. Regarding hand washing habits, 13 % of mothers from irrigation users and 5.2% of mothers from non-irrigation users use always soap and water (Table 1).

### Prevalence of stunting and other characteristics

The overall prevalence of stunting was 36.5% (95% CI 33.8 to 39.3%) and did not show significant variation between irrigated 32.8% (95%CI 29.1 to 36.7%) and non-irrigated users 40.2% (95%CI 36.3 to 44.2%). Sixty-three percent (63.4%) of children from irrigation users and 57.9% of children from non-irrigation users had initiation breast feed within one hour. Half of children (50.2%) from irrigation users and 54.0% of mothers from non-irrigation users were male by sex (Table 2).

**Table 2:** Prevalence of stunting and diarrhea, and Height for Age among irrigation and non-irrigation users in Mecha district, Northwest Ethiopia, 2019

Characteristics	Irrigated		Non-irrigated		Total	Chi-square (***p***-value)
	Fre.	percent	Fre.	percent		
**Child Stunting status**						
Stunted	191	32.8	234	40.2	425 (36.5)	6.85 (0.009)
Not stunted	391	67.2	348	59.8	739 (63.5)	
**Initiation of Breast-feeding**						
Within 1 h	369	63.4	337	57.9	706 (60.7)	3.69 (0.05)
More than 1 h	213	36.6	245	42.1	458 (39.3)	
**Age start complementary feeding**						
≥ 6 months	518	89.0	430	73.9	948 (81.4)	44.02(< 0.001)
< 6 months	64	11.0	152	26.1	216 (18.6)	
**Child sex**						
Male	292	50.2	314	54.0	606 (52.1)	1.67 (0.197)
Female	290	49.8	268	46.0	558 (47.9)	
**Child age**						
< 12 months	33	5.7	86	14.8	119 (10.2)	65.66(< 0.001)
12–23 months	81	13.9	119	20.4	200 (17.2)	
24–35 months	143	24.6	175	30.1	318 (27.3)	
36–47 months	166	28.5	119	20.4	285 (24.5)	
48–60 months	159	27.3	83	14.3	242 (20.8)	
**Had diarrhea**						
Yes	99	17.0	119	20.4	218 (18.7)	2.26 (0.133)
No	483	83.0	463	79.6	946 (81.3)	
**Had Fever**						
Yes	105	18.0	132	22.7	237 (20.4)	3.86 (0.049)
No	477	82.0	450	77.3	927 (79.6)	

Nearly one fourth of children (24.6%) from irrigation users and 30.1% of mothers from non-irrigation users were between 24 and 35 months.. Eighteen percent of children (24.6%) from irrigation users and 22.7% of mothers from non-irrigation users had fever in the past 2 weeks before preceding the survey (Table 2).

### Factors associated with stunting in irrigation and non-irrigation users

Based on the results of the multivariable logistic regression model, the odds of stunting are associated with ANC visit. The odds of child stunting from a mother that did not visit ANC were 2.61 [AOR = 2.61; 95% CI: 1.33–5.12] higher compared to a child from a mother who had more than three visits. The odds of child stunting whose age between 12 and 23 months, 24 and 35 months, and 36 and 47 months were 2.60 times [AOR = 2.60; 95% CI: 1.51–4.48], 2.92 times [AOR = 2.92; 95% CI: 1.74–4.89], and 3.55 times [AOR = 3.55; 95% CI: 2.09–6.01], higher compared to a child whose age less than 12 months respectively. The odds of child stunting from a respondent who washed their hands with water only and sometimes with soap and water were 2.27 times [AOR = 2.27.; 95% CI: 1.25–4.22] and 1.77 times [AOR = 1.77.; 95% CI: 1.06–2.97] higher compared to a respondents who always practicing hand washing with soap and water. The odds of stunting in a child who had fever were 1.77 times [AOR = 1.77; 95% CI: 1.29–2.43] higher compared to a child who had no fever for the last 2 weeks before preceding the study (Table 3).

**Table 3:** Multivariable Logistic Regression Analysis of factors affecting childhood stunting in irrigation and non-irrigation users in Mecha district, Northwest Ethiopia, 2019

Characteristics	Stunting		COR (95%CI)	AOR (95%CI)
	Yes	No		
**Irrigation status**				
Irrigated	**191**	**391**	1.00	
Non-irrigated	234	348	1.37 (1.08–1.74)**	
**Child Sex**				
Male	227	379	1.09 (0.86–1.38)	
Female	198	360	1.00	
**Women’s age**				
< 20 years	10	23	1.00	
20–29 years	118	221	1.23 (0.57–2.67)	
≥ 30 years	297	495	1.38 (0.65–2.94)	
**Family size**				
≤ 4 members	111	213	1.00	
> 4 members	314	526	1.15 (0.87–1.50)	
**Women education**				
Unable to read and write	365	589	1.55 (1.12–2.15)**	
Able to read and write	60	150	1.00	
**Initiation of Breast-feeding**				
Within 1 h	262	444	1.06 (0.84–1.36)	
More than 1 h	163	295	1.00	
**Age start complementary feeding**				
≥ 6 months	338	610	1.00	
< 6 months	87	129	1.22 (0.90–1.65)	
**Antenatal care visit**				
No visit	29	17	3.68 (1.96–6.92)***	2.61 (1.33–5.12)**
1–3 visits	144	311	1.32 (1.03–1.70)*	1.21 (0.93–1.57)
> 3 visits	252	411	1.00	1.00
**Frequency of PNC**				
No visit	376	620	1.54 (0.76–3.13)	
1–2 visit	38	91	1.07 (0.48–2.35)	
> 3 visit	11	28	1.00	
**Heath facility far from home**				
< 1 h	214	435	1.00	
> =1 h	211	304	1.41 (1.10–1.79)**	
**Child sex**				
Male	227	379	1.00	
Female	198	360	0.92 (0.72–1.17)	
**Child age**				
< 12 months	24	95	1.00	1.00
12–23 months	77	123	2.48 (1.46–4.21)**	2.60 (1.51–4.48)**
24–35 months	132	186	2.81 (1.70–4.63)***	2.92 (1.74–4.89)***
36–47 months	131	154	3.38 (2.03–5.58)***	3.55 (2.09–6.01)***
48–60 months	61	181	1.33 (0.78–2.27)	1.39 (0.80–2.42)
**Drinking water source**				
Improved source	311	557	1.00	
Unimproved source	114	182	1.12 (0.85–1.47)	
**Latrine facility**				
Improved source	211	405	1.00	
Unimproved source	214	334	1.23 (0.97–1.56)	
**Ways of Hand washing habits**				
Only with water	105	129	3.14 (1.84–5.37)***	2.27 (1.25–4.11)**
Sometime with soap and water	298	525	2.19 (1.34–3.58)**	1.77 (1.06–2.97)*
Always with soap and water	22	85	1.00	1.00
**Had diarrhea**				
Yes	94	124	1.41 (1.04–1.90)*	
No	331	615	1.00	
**Had Fever**				
Yes	113	124	1.80 (1.35–2.40)***	1.77 (1.29–2.43)***
No	312	615	1.00	1.00

*P < 0.001, **P < 0.01, ***p < 0.05

## Discussion

The study revealed that the overall prevalence of stunting is 36.5% (95%CI 33.8 to 39.3%) among the participating HHs. This finding is consistent with the mini DHS result in Amhara Region (42%) [20], East and West Gojjam zone of Amhara region (37.5%) [21]. However, this result is lower than the prevalence reported from the previous study conducted in West Gojjam (43.5%) [22] and in Awi Zone (50%), Amhara region [23]. According to the WHO’s classification, the prevalence of stunting in the study area is high (> 40% is very high, 30 to 39% is high, 20 to 29% is medium and < 20% is classified as a low prevalence [24]. The variation in the prevalence of stunting in different studies may be attributed to a broad range of interrelated factors such as environmental or geographical factors (e.g. population density, rainfall and temperature variation and disease environment), socio-cultural differences in relation to child feeding and care practices, maternal under nutrition, HH food insecurity, economic growth and maternal education [21, 25–27].

This study found that significant variation of child stunting did not observe between irrigated and non-irrigated user. The finding of the study is inconsistent with previous studies, which showed that irrigation is associated with a reduced risk of stunting [28, 29]. Studies revealed that irrigation schemes can ensure household food security [28, 29]. Although food security may be a necessary prerequisite for good nutrition outcomes, it is insufficient by its own to improve nutritional outcomes [30]. The nutritional status of children can be addressed by adopting a multi-sectorial approach [31, 32] and determined by mother knowledge on child nutrition practice, maternal nutritional status, intra household food allocation and utilization practices, and access to health services and healthy environmental conditions. For example, the head of the family gets first priority in eating while mothers and children get a smaller share of the family’s food relative to their nutritional need due to hierarchical position with intra-family food distribution [33]. A study also revealed that some farmers using irrigation schemes engaged in market-oriented crops (Nkonya E, Iannotti L, Sakwa B, Wielgosz B, Gandhi V, Kato E, et al: Baseline study of KickStart treadle pumps in East Africa, unpublished) which might not be necessary improve the nutritional status of children. Thus, further study is recommended to clearly understand the root cause of child malnutrition in the study area community.

Children aged > 12 months were found a significant association with child stunting compared to infants of less than one year in this study which is consistent with other studies [34, 35]. The extent of stunting usually common after age of 12 months due to several reasons. The children usually terminate breastfeeding after age of 12 months, do not get adequate diet and proper care, and expose to pathogens due to poor environment facilities at household level such as house with poor hygiene, unsafe water supply and sanitation [36]. Literature indicates improved behavioral practices such as hand washing before eating early on are important to consider along with other factors closely associated with the age of the child [34].

In this study, children whose mother had no ANC visit increase the odds of child stunting compared to multiple contacts during ANC visits which is consistent with previous studies [37, 38]. Mothers who made frequent and regular contacts with health care provider get a chance for interactive health education sessions. Thus, multiple contacts during ANC enhance mother knowledge of appropriate feeding for their infants after delivery, including breastfeeding and feeding a diverse diet, and practice preventive and curative child care activities such as timely seeking of health care services, which in turn can have a positive effect on children’s nutritional status.

The result of this study indicated that male children were slightly more stunted than female children (53 and 47%, respectively) but not at a level of statistical significance, which is consistent with other findings [39, 40]. There are studies which show males are at higher risk for stunting [41–44] whereas another study found that females are at higher risk for stunting [45]. These sex-related differences may require further study to understand the relationship between child sex and nutrition.

In this study, improved water supply and sanitation did not show a significant reduction in child stunting, however, hand washing practice with soap was one of the significant factors associated with a reduction in child stunting. Inconsistent findings on benefits of improved water supply and sanitation and hygiene in child stunting reduction were reported [34, 46, 47]. Some studies showed a strong association between the level of environmental WASH (Water, Sanitation and Hygiene) and child linear growth [36, 48]. The concept of a clean play and infant feeding area as an additional component of early child development programs along with WASH is one of the recommended strategies to reduce child stunting [34, 36, 48–50]. The literature indicates that sanitation and water supply improvements, hand washing with soap, ensuring a clean play and infant feeding environment and food hygiene are all important to interrupt the multiple transmission pathways of fecal-oral pathogens [36, 49]. Better hygiene practices are essential to prevent diarrhea and other infections among children, which in turn contribute to reducing child stunting.

Previous studies on the association between improved drinking water stunting found inconsistent findings, while other studies have shown that the definition of “improved” does not reliably predict the microbiological safety of the water [51, 52]. Studies revealed that household access to an improved source of drinking water or piped water was not associated with child stunting [53, 54]. On the other hand, a study done by Fink et al. using the merged data set of 171 Demographic and Health Surveys found access to improved drinking water was associated with a lower risk of mild or severe stunting [55]. There is inconsistent findings of synergistic effects of improved drinking water and sanitation on childhood stunting [35, 56, 57]. The possible reasons for this variation may be related with initial water quality at the source, and with water handling practices during collection, transport and storage at the household level. There is evidence that improved water sources do not correlate well with water that is safe for human consumption [58, 59] and that water quality deteriorates from source to point of consumption due to unsafe collection, transport, storage and handling practices [60, 61]. Thus, further research is required to determine if an improved water source that fulfills the recommended quality standard for consumption, along with safe drinking water handling and storage practices, and the combined effect with improved sanitation, have independent or synergistic effects on child stunting.

The study has strengths. One strength is that this study employed multivariable logistic regression models to control confounding effect and determine the independent effect of irrigation users on child stunting.

### Limitations of the study

The study has limitations. There might be a social desirability bias and a recall bias during answering of questions related to hygienic practices and events happening in the past, such as the child’s history of illness and child care practices. Moreover, information on some important confounding variables such as mothers’ nutritional status during pregnancy, parasitic infection and the child’s birth weight were not collected, and these could also have influenced children’s nutritional status. Interpretations of factors associated with child stunting should be with caution since a cause and effect relationship cannot be established using a cross-sectional study design.

## Conclusions

In conclusion, there is a high prevalence of stunting in children from 6 to 59 months in the study area, with no variation between irrigation and non-irrigation users. Mothers who had no ANC visit, children whose age was between 12 and 47 months, mothers who did not practice hand washing with soap always and children who had fever were the factors associated with higher child stunting. As the government of Ethiopia confront to reduce food insecurity and child stunting through irrigation practices. This study provides insight that irrigation only may not sufficient to improve the nutritional status of children. Therefore, decision makers and program implementers may benefit from the findings of this study to revise strategies and integrate agricultural practice with nutrition education. Strengthening implementation of the government’s agricultural practice, combined with interventions to empower women during ANC visit, could accelerate further reductions in child stunting.

## Supplementary Information

**Additional file 1.**

## Data Availability

The data set used for final analysis will be available upon corresponding author request.

## Abbreviations

AOR: Adjusted Odds Ratio
CI: Confidence Interval
COR: Crude Odds Ratio
SD: Standard Deviations
WHO: World Health Organization
