# Supplementary material for: Childhood stunting and associated factors among irrigation and non-irrigation user northwest, Ethiopia: a comparative cross-sectional study
Source: Ital J Pediatr. 2021 Apr 26;47:102. doi: 10.1186/s13052-021-01048-x (PMC8074471; doi:10.1186/s13052-021-01048-x)
Supplement: Supplementary file 1 — Additional file 1. [file 13052_2021_1048_MOESM1_ESM.docx]

Schematic Presentation of Sampling Procedure

**From Mecha District**

**(kebele were selected by SRS**)

**Non- Irrigated kebele (6 of 30)**

**9,335 HH**

**Irrigated kebele (6 of 10)**

**9,593 HH**

After proportional

Allocation done by SS

**(582)**

**HH**

**(582)**

**HH**

Distributed to their

Kebele

**F**

**B**

**A**

**C**

**D**

**E**

**B**

**F**

**C**

**D**

**A**

**E**

Key (name of kebele & sample) Key (name of kebele & sample)

A= Kudmi (138) A= Mekeni (122)

B= Ediget Behibret (93) B=Enamirt (118)

C= kolella (66) C=Tatek lesira (117)

D=Agamina (59) D=Midregenet (108)

E=Tekle Dib (99) E=Deremeni (35)

F_=_ Ambo mesik (127)  F=Birakat Zuria (82)
